# Antibacterial Activity against *Escherichia coli* and Cytotoxicity of Maillard Reaction Product of Chitosan and Glucosamine Prepared by Gamma Co-60 Ray Irradiation

**DOI:** 10.3390/polym15224397

**Published:** 2023-11-13

**Authors:** Anh Quoc Le, Van Phu Dang, Ngoc Duy Nguyen, Chi Thuan Nguyen, Quoc Hien Nguyen

**Affiliations:** 1 Faculty of Biology-Biotechnology, University of Science, Ho Chi Minh City 700000, Vietnam; anhquoc1704@gmail.com; 2 Vietnam National University, Ho Chi Minh City 700000, Vietnam; 3 Research and Development Center for Radiation Technology, Vietnam Atomic Energy Institute, Ho Chi Minh City 700000, Vietnam; 4 Vietnam Atomic Energy Institute, Hanoi 100000, Vietnam

**Keywords:** chitosan, glucosamine, Maillard reaction, antibacterial effect, cytotoxicity

## Abstract

In this study, the gamma ray-induced Maillard reaction method was carried out for chitosan (CTS) and glucosamine (GA) to improve the water solubility and antibacterial activity. The mixture solution of CTS and GA was exposed to gamma rays at a dose of 25 kGy and freeze-dried to obtain a Maillard reaction product (MRP) powder. The physicochemical and biological properties of the CTS-GA MRP powder were investigated. The CTS-GA MRP powder expressed good solubility at a concentration of 0.05 g/mL. In addition, the result of the antibacterial activity test against *Escherichia coli* revealed that the CTS-GA MRP powder exhibited highly antibacterial activity at pH 7; in particular, bacterial density was reduced by over 4 logs. Furthermore, the cytotoxicity test of the CTS-GA MRP powder on mouse fibroblast cells (L929) showed non-cytotoxicity with high cell viability (>90%) at concentrations of 0.1–1 mg/mL. Owing to the high antibacterial activity and low cytotoxicity, the water-soluble CTS-GA MRP powder can be used as a favorable natural preservative for food and cosmetics.

## 1. Introduction

Food safety is a topic that has never run out of steam. The World Health Organization (WHO) estimated that unsafe food caused 600 million cases of foodborne diseases and 420,000 deaths each year between 2007 and 2015 [1]. Furthermore, food waste and food loss are other challenges threatening food security. Every year, 1.3 billion tons, or one-third of total global food production, is lost or wasted, which is estimated at about USD 1 trillion [2]. Hence, developing a new preservative to prevent foodborne illness, as well as to reduce food waste, could save billions of US dollars every year for the world’s economy. Because of the growing awareness and concern regarding food safety and the harm of chemical and synthetic preservatives, alternative strategies for food preservatives are required, and natural additives are arguably the most promising suggestion.

Chitosan (CTS), a polycationic biopolymer derived through the alkaline deacetylation of chitin, is mainly produced from shellfish-processing waste [3,4]. Besides some characteristics such as being biodegradable, non-allergenic, and nontoxic, CTS also exhibits versatile biological activities such as antioxidative, antimicrobial, and anticancer activity. Hence, CTS and its derivatives have gained much interest as a potential food preservative of natural origin [5]. Although the antibacterial mechanism of CTS is still inconclusive, three proposals receive the most consensus: (1) CTS interacts with macromolecules on the bacterial cell wall by electrostatic adsorption and alters its permeability; (2) CTS enters the inside of bacterial cells, binds to DNA, and causes the inhibition of RNA and protein synthesis; and (3) CTS forms a chelate with essential nutrients for cell growth [6]. In terms of safety, CTS derived from shrimp shells achieved GRAS (Generally Recognized as Safe) status from the Food and Agriculture Organization of the United Nations (US-FDA) in 2001 and thus broadened its application objects for food preservation purposes [7], including fruit and vegetables [8], seafood [9], meat, and meat products [10]. However, the application of CTS in many fields is still restricted because of its insolubility in water as well as a reduction in biological activities at neutral or basic pH [3]. CTS derivatives with good solubility in water can be easily applied in many fields. Therefore, there have been many efforts to improve the solubility and biological activities of CTS based on chemical or enzymatic modifications, in which chemical modifications are generally not preferred in food applications [4]. 

The Maillard reaction (MR) is usually known as a non-enzymatic browning reaction between the carbonyl groups of reducing ends in carbohydrates and the amino groups of amino acids, proteins, or any nitrogenous compounds by heating or irradiating [11]. The MR is a very complex reaction that occurs spontaneously during thermal food processing and produces a wide range of Maillard reaction products (MRPs), which effectively contribute to the flavor formation and antibacterial and antioxidant activities of foods [11,12]. It is generally agreed that there is a substantial amount of MRPs in the average human diet [13]. Therefore, this reaction is considered a friendly green method to improve the properties of CTS for food applications. In the last decade, numerous studies have investigated the effect of CTS–sugar MRPs to increase the nutritional qualities of food as well as to extend its shelf life. The heating-induced MRPs of CTS or its derivatives with saccharides or proteins have been reported to be promising preservative agents for many kinds of food, such as fish, seafood, meats, and noodles, due to their good solubility and bacterial activity. In fact, the MR not only incorporates hydrophilic groups such as the hydroxyl group of monosaccharide onto the CTS chain to improve its solubility but also preserves its global properties, as well as enhances some biological activities. Among the Maillard derivatives of CTS with various saccharides, the one of CTS with GA, which contains both a hydroxyl group and an active amino group, exhibits good solubility in a relatively wide pH range and excellent antibacterial activities [5,14]. Recently, it has been recorded that gamma-ray irradiation could induce Maillard reactions like heating [15,16]. More interestingly, the gamma ray-induced MR can take place rapidly at room temperature without forming any toxic byproducts, such as 5-hydroxymethylfurfural [17]. However, up to now, the number of publications on the preparation of gamma ray-induced MRPs of chitosan–glucosamine, especially for food applications, is still limited. In this study, the CTS-GA MRP solution was prepared using a gamma Co-60 ray irradiation method. The solution was freeze-dried to obtain CTS-GA MRP powder. Its solubility in water was determined. Furthermore, the antibacterial activity against *Escherichia coli* and cytotoxicity on mouse fibroblast cells (L929) of CTS-GA MRP powder were also investigated.

## 2. Materials and Methods

### 2.1. Materials 

Chitosan from shrimp shell with an average molecular weight (Mw) of ~97 ± 5 kDa and a degree of deacetylation of ~90 ± 3% was supplied by Sun Eco Green Import Export Company Limited, Ho Chi Minh City, Vietnam. Glucosamine was purchased from Merck (Darmstadt, Germany). The *Escherichia coli* ATCC 51813 was provided by the Metabolic Biology Laboratory, University of Science, Ho Chi Minh City. The Mueller–Hinton medium and agar were purchased from Himedia Laboratories Private Limited, Thane, India. Other chemicals such as lactic acid, NaOH of analytical grade, and distilled water were used for all experiments.

### 2.2. Preparation of CTS-GA MRPs

The preparation of CTS-GA solutions was carried out according to the method described in our previous research [18]. Briefly, a solution of 2% (*w*/*v*) CTS in 1% (*v*/*v*) lactic acid was prepared. Similarly, a 2% solution of GA in distilled water was also prepared. Four volumes of 2% CTS solution were mixed with one volume of 2% GA solution to obtain the mixture of CTS (1.6%) and GA (0.4%). The CTS-GA mixture solution was exposed to γ-ray from a Co-60 source with a dose of 25 kGy at a dose rate of 1.3 kGy/h with a Gamma-cell 5000 (BRIT, Mumbai, India) to perform a Maillard reaction. Consequently, the irradiated solution was freeze-dried to obtain CTS-GA MRP powder. To confirm the formation of Maillard reaction product, UV-Vis spectra of CTS-GA MRP solution were obtained with a spectrophotometer (Jasco-V630, Tokyo, Japan) at wavelengths of 284 nm and 420 nm [19]. Furthermore, the free GA content in the solution before and after irradiation was determined using the high-performance liquid chromatography (HPLC) method according to AOAC 2012 (2005.01) on an Agilent 1200 series Infinity with a UV-Vis detector (Agilent, Santa Clara, CA, USA). The efficiency of the Maillard reaction was expressed as the ratio of reacted GA to the initial GA by Equation (1) [18]:

Maillard reaction efficiency (%) = [(M_0_ − M_t_)/M_0_] × 100 (1)

where M_0_ and M_t_ are the free GA content of the CTS-GA solution before and after irradiation, respectively.

### 2.3. Determination of Solubility in Water

Firstly, CTS-GA MRP powder was dissolved in 10 mL water for the final concentration from 0.01 to 0.05 g/mL by stirring for 5 h at room temperature. Afterward, the solutions were filtered through 0.45 μm filter paper, which was dried in a forced-air oven at 60 °C until constant weight. The solubility was calculated from the change in the filter paper’s weight [14]. The preparation of CTS powder from 25 kGy irradiated CTS solution (iCTS) was performed similarly. The solubility in water of the samples was calculated with Equation (2):

Solubility (%) = [1 − (M_Ft_ − M_F0_)/M_sample_] × 100 (2)

where M_Ft_ and M_F0_ are the weight of the filter after and before filtering; M_sample_ is the weight of sample that was dissolved in water.

### 2.4. Fourier-Transform Infrared (FTIR) Spectroscopy Analysis

FTIR spectra were recorded with an NIR MIR Frontier (Perkin Elmer, Waltham, MA, USA) by scanning along a spectrum range of 400–4000 cm^−1^. The CTS or CTS-GA MRP powder was ground with KBr in a ratio of 1/100 (1 mg sample with 100 mg KBr) and then compressed to form discs [12]. 

### 2.5. Proton Nuclear Magnetic Resonance (^1^H-NMR) Analysis

^1^H-NMR analysis was performed on a Brucker Avance 500 MHz at 70 °C. The CTS or CTS-GA MRP powder was dissolved in D_2_O solution to obtain a polymer concentration of 5 mg/mL, and measurements were carried out at 70 °C [12].

### 2.6. Detection of 5-Hydroxylmethylfurfural

The presence of 5-hydroxylmethylfurfural (5-HMF) in the CTS-GA MRP powder was detected using reverse-phase HPLC with the method described by Theobald et al. (1998) [20]. Chromatography analyses were carried out on an Agilent 1260 infinity HPLC system with a UV-Vis detector at 284 nm and a Restek Ultra Aqueous C_18_ column (250 mm × 4.6 mm). The CTS-GA MRP powder was dissolved in water and filtered through a 0.45 μm PTFE membrane. The injection volume was 10 μL, and the run time was 20 min at room temperature. A mixture of acetonitrile/water (15/85, *v*/*v*) was used as a mobile phase with a flow rate of 1 mL/min. A 5-HMF solution (20 μg/mL) was used as a standard, and the CTS-GA solution heated at 65 °C for 5 days was used to compare with the MRP prepared with the gamma-ray irradiation method [3].

### 2.7. Evaluation of Antibacterial Activity

The antibacterial activity of the CTS-GA MRP was investigated against *E. coli* ATCC 51813 using the disk diffusion method and poisoned food method described by Balouiri et al. (2016), with some modifications [21]. In the disk diffusion test, a Mueller–Hinton agar plate was spread with *E. coli* (~10^4^ CFU/mL). Afterward, filter paper discs (about 6 mm in diameter) coated with the powder of CTS, iCTS, and CTS-GA MRP were placed on the plate. A blank disc was also placed on the plate to serve as a control. Then, the plate was incubated overnight at 37 °C and colony formation was monitored.

In the poisoned food test, 0.04 g the powder of iCTS or CTS-GA MRP was added into a flask containing 99 mL of Mueller–Hinton broth medium at pH 7. Afterward, 1 mL of the bacterial suspension (~10^8^ CFU/mL) was aseptically inoculated into each flask. And then, the flasks were shaken at 150 rpm for 4 h at room temperature. A flask containing only broth and bacterial suspension was established in parallel to serve as the control. After shaking, the survival cell density in each mixture was determined using the spread plate technique [21]. The antimicrobial activity of samples was expressed by the reduction in bacterial density (log CFU/mL) in the testing samples compared with the control.

### 2.8. Evaluation of Cytotoxicity

The cytotoxicity of the CTS-GA MRP was estimated using a 3-(4,5-dimethyl-2-thiazolyl)-2,5-diphenyl-2H-tetrazolium chloride (MTT) viability assay, as described by Zhang et al. (2016), with slight modifications [22]. The L939 cells were cultured in Dulbecco’s Modified Eagle’s Medium (DMEM) supplemented with 10% BSA (*w*/*v*) and 100 U/mL of antibiotic. Cells were grown in 96-well plates at 37 °C in a humidified atmosphere (5% CO_2_) for a density of 1 × 10^4^ cells/well. After 24 h, the cells were treated with a medium supplemented with the CTS-GA MRP for the final concentrations of 0.1–1.0 mg/mL. The cells treated with DMEM medium or DMSO 20% served as negative or positive controls, respectively. All samples were incubated for 24 h, and the culture of each well was replaced by a 100 μL aliquot of MTT solution (1 mg/mL) and incubated for 4 h. Then, the supernatant in each well was aspirated and replaced by a 100 μL aliquot of DMSO/ethanol (1/1) solution to solubilize formazan crystals. The absorbance at 540 nm (OD540) of the wells was measured using an ELISA plate reader EZ Read 400 (Biochrom, Cambourne, UK). The relative growth rate (RGR) of cells treated with the CTS-GA MRP in the tested concentration range was determined according to Equation (3) [22]:

RGR (%) = (OD540_test sample_/OD540_negative control_) × 100 (3)

### 2.9. Statistical Analysis

Data of UV-Vis spectra measurements and cytotoxicity evaluation were expressed as the mean ± standard error (SE). One-way ANOVA was performed for each sample, including three replicates. Significant differences between means were determined with the Turkey test at a 0.05 probability level (*p* < 0.05).

## 3. Results

### 3.1. Preparation of the MRPs and UV-Vis Spectrophotometric Analyses

**Figure 1:**
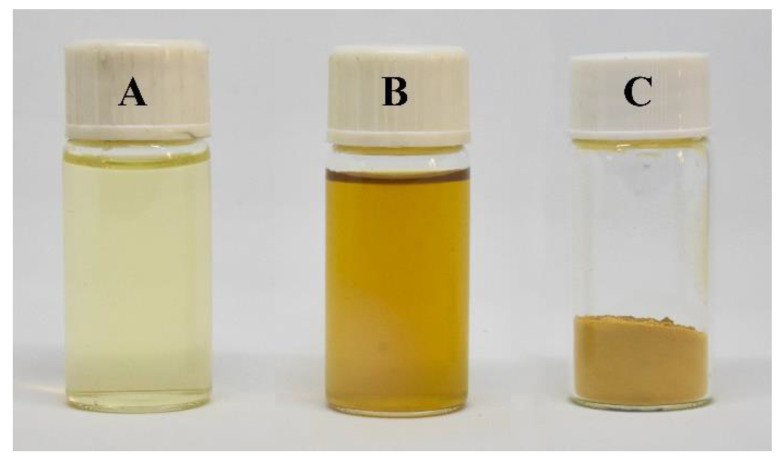
The visual color of the CTS-GA solution (**A**), 25 kGy irradiated CTS-GA solution (**B**), and CTS-GA MRP powder (**C**).

The visual colors of the CTS-GA solution before/after irradiation and CTS-GA MRP powder are presented in Figure 1. The change in color of the CTS-GA solution was easily observed after irradiation, namely, the color of the solution became browner. Moreover, this color change was also expressed by the results of the absorbance measurements in Table 1. The absorbance intensities at 284 and 420 nm were increased significantly after irradiation. The same results were also recorded in other studies where the protein–sugar solutions were treated by heating [4] or irradiating [10,16]. In the Maillard reaction, the intermediate stage products can be detected by UV absorbance at 284 nm, while absorbance at 420 nm is preferred for the final stage products [15,19]. Furthermore, upon irradiation, the free GA content in the solution decreased significantly from 4.05 to 0.84 ± 0.09 mg/mL, corresponding to a reaction efficiency of 79.28% (Table 1). Therefore, the results confirmed that MRPs were formed effectively with 25 kGy irradiation.

### 3.2. Solubility of CTS-GA MRP Powder in Water

**Figure 2:**
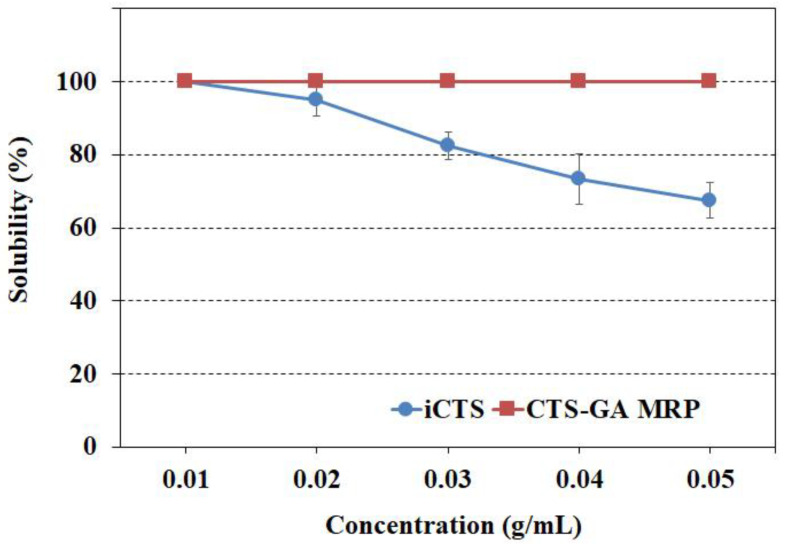
The solubility of iCTS and CTS-GA MRP powder in water.

The solubility of the iCTS and CTS-GA MRP powders at various concentrations in water is shown in Figure 2. At a concentration of 0.01 g/mL, both powders were completely dissolved. Along with the increase in the concentration, the solubility of the iCTS powder gradually decreased, whereas the solubility of the CTS-GA MRP powder still remained at 100% up to 0.05 g/mL. This result indicated that the MR effectively improved the solubility of CTS. This can be attributed to the increase in the hydroxyl groups in the CTS chains and the decrease in hydrophobic intermolecular interactions that favor polymer aggregation. The same phenomena were also obtained in previous studies [3,23]. 

### 3.3. FTIR Spectroscopy

**Figure 3:**
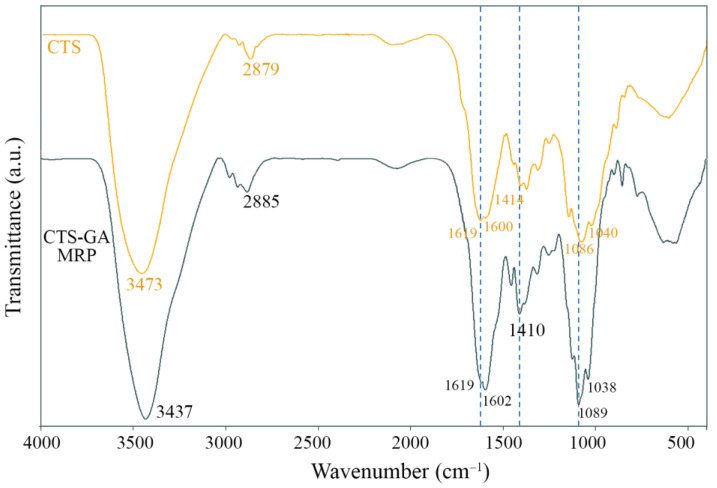
FTIR spectra of CTS and CTS-GA MRP.

The FTIR spectra of CTS and CTS-GA MRP in the range from 4000 to 400 cm^−1^ are presented in Figure 3. The characteristic bands can be observed at 3473 cm^−1^ (O–H and N–H stretching), 2879 cm^−1^ (C–H stretching vibration), 1619 cm^−1^ (C=O stretching vibrations, amide I), 1600 cm^−1^ (amide II), 1414 cm^−1^ (C–H_2_ stretching), and 1086 and 1040 cm^−1^ (C–O stretching) on the CTS pattern [24,25]. After irradiation, these bands of CTS were changed. In the spectrum of the CTS-GA MRP, the band at 1619 cm^−1^ shifted to 1602 cm^−1^, suggesting that Schiff base (C=N double bond) was formed between the carbonyl groups of GA and the amino groups of CTS. In addition, the band at 1414 cm^−1^ shifted to 1410 cm^−1^ in the spectrum of the CTS-GA MRP, indicating an increase in the number of –CH_2_ groups due to the introduction of GA into the CTS molecule. Similar results were also recorded in other studies [12,24]. Furthermore, the spectrum of the CTS-GA MRP also showed the bands at 1089 cm^−1^ and 1038 cm^−1^ assigned to the C–O stretching vibration. In the region of 1200–400 cm^−1^, the variation in the bands in the CTS-GA MRP spectrum was more pronounced than that in CTS [12]. These changes demonstrated that the structure of CTS was modified by gamma ray-induced MR.

### 3.4. ^1^H-NMR Spectroscopy

**Figure 4:**
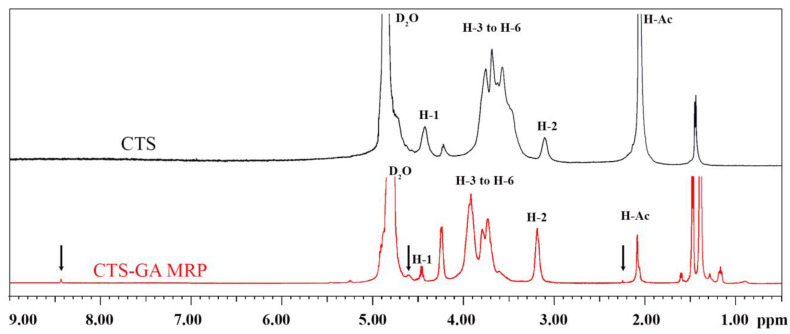
^1^H-NMR spectra of CTS and CTS-GA MRP.

^1^H-NMR analysis was used to confirm the introduction of the GA unit into the CTS molecule. The ^1^H-NMR spectra of CTS and CTS-GA MRP are displayed in Figure 4. The characteristic signals in the CTS spectrum were observed. The peak at δ 2.0 ppm is assigned to the methyl protons of N-acetyl glucosamine (H-Ac). The peak at δ 3.1 ppm belongs to the H-2 proton of the GA ring. The multiplet at δ 3.5–4.0 ppm is attributed to the H-3–H-6 protons of the pyranose ring. The signal at δ 4.4 ppm represents the H-1 proton of N-acetyl glucosamine (H-1A), and the other one at δ 4.7–4.9 ppm is the H-1 proton of GA (H-1D) that was overlapped by the strong signal at δ 4.8 ppm of the solvent (D_2_O) [25,26].

By comparing the ^1^H-NMR spectra, some differences between CTS and CTS-GA MRP were found. In particular, a new signal for the –N=CH– group (Schiff base), an intermediate product of MR, was observed at δ 8.45 ppm in the CTS-GA MRP spectrum. In addition, a new signal appeared at δ 2.25 ppm, corresponding to the –CH_2_ of GA linked to the –NH_2_ of CTS, indicating a displacement of the –N=CH– linkage (Schiff base) toward –NH–CH_2_– [12,23]. A new signal at δ 1.6 ppm may be assigned to the proton of the alkyl group [25]. Furthermore, in the region of δ 4.5–4.6 ppm, some signals were also observed, indicating the N-substitution of the –NH_2_ groups of CTS [12,25]. The new peaks in the region of δ 1.3–1 ppm in the CTS-GA MRP spectrum in Figure 4 are still unknown. Therefore, the changes in the ^1^H-NMR spectrum in Figure 4 revealed the alteration in the structure of CTS by the binding of GA to the CTS backbone due to gamma ray-induced MR.

### 3.5. Detection of 5-Hydroxylmethylfurfural

**Figure 5:**
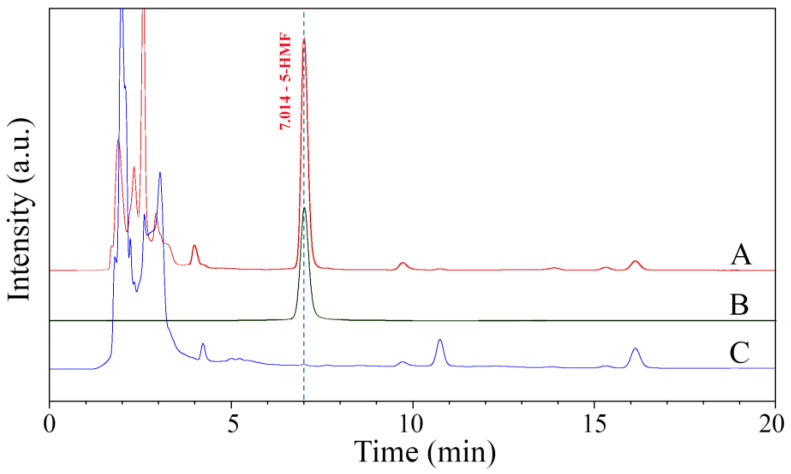
HPLC chromatograms of heat-induced CTS-GA MRP (A), 5-HMF standard (B), and gamma ray-induced CTS-GA MRP (C).

5-Hydroxylmethylfurfural (5-HMF) is an intermediate product of the Maillard reaction [27]. It is cytotoxic at high concentrations and irritating to the eyes, upper respiratory tract, skin, and mucous membranes; the oral LD_50_ value in rats was determined to be 3.1 g/kg body weight [28]. Under the chromatographic HPLC conditions described above, the retention time of the 5-HMF peak in the standard solution appeared at about 7.0 min (Figure 5). It was also observed in Figure 5 that a characteristic peak of 5-HMF appeared in the heat-induced CTS-GA MRP chromatogram which was not detected in the gamma ray-induced CTS-GA MRP chromatogram. This result indicated that the gamma-ray irradiation method could induce the MR between CTS and GA without forming 5-HMF. The same results were also reported in previous research [16,17]. Oh et al. (2005) [16] reported that no furfurals were detected in irradiated sugar–amino acid solution, whereas these compounds were found in heated solutions. 

### 3.6. Antibacterial Activity

**Figure 6:**
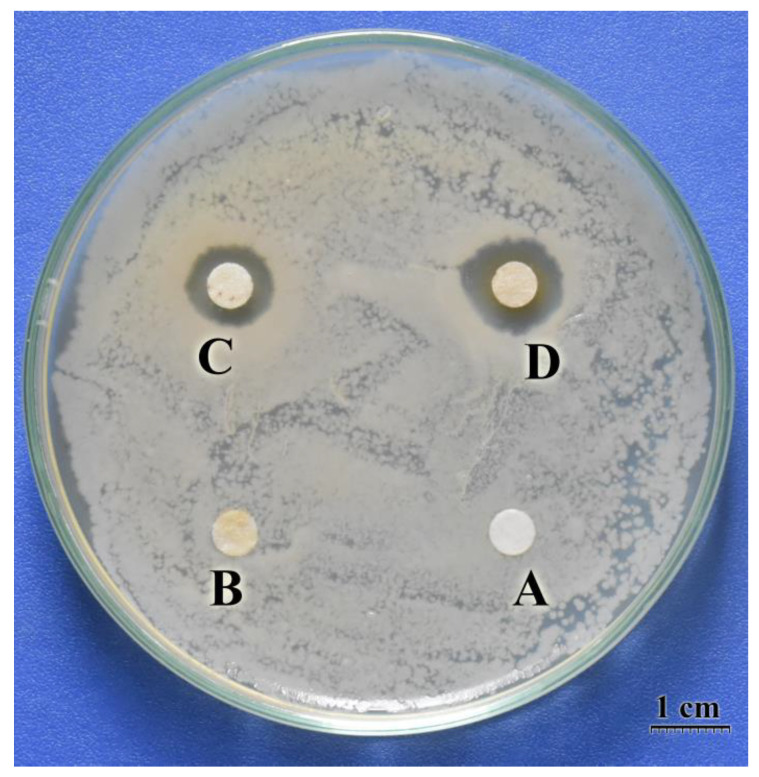
Photograph of the antibacterial activity assessed using the disk diffusion method. (A) Control; (B) CTS powder; (C) iCTS powder; and (D) CTS-GA MRP powder.

The results in Figure 6 indicated that both iCTS and CTS-GA MRP disks were able to form an inhibition zone on the *E. coli* plate, while the control and CTS disks were not. This result may be attributed to the solubility of the powder on paper disks. On the *E. coli* plate, paper disks absorbed moisture and dissolved the powders. Because of the poor solubility in water, CTS powder did not show an antibacterial activity. On the other hand, the iCTS and CTS-GA MRP powders were well soluble in water (see Figure 2), so they exhibited highly antibacterial activities. In addition, the antibacterial activity of these powders could be evaluated by the diameter of the inhibition zones [21]. Therefore, the antibacterial activity against *E. coli* of the CTS-GA MRP powder was estimated to be higher than that of the iCTS powder. 

**Figure 7:**
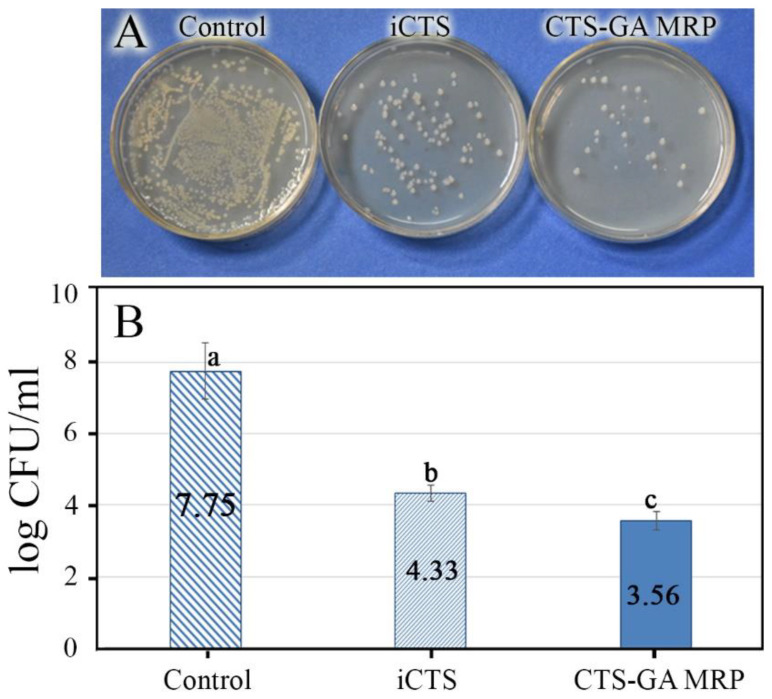
Viable bacterial colonies at the same dilution of 10^−2^ (**A**) and bacterial density (**B**) of the control, iCTS, and CTS-GA MRP samples. The mean values in (**B**) with different letters are significantly different (*p* < 0.05) according to the Tukey test.

The results in Figure 7 indicated that after 4 h of shaking, the bacterial density in both agents of the iCTS and CTS-GA MRP was significantly decreased in comparison with that of the control. It has been shown that the lower the density of the viable bacteria, the higher the antibacterial activity. Furthermore, the results also revealed that the antibacterial activity of the CTS-GA MRP was higher than that of iCTS. Compared to the control, the CTS-GA MRP reduced the bacterial density by up to over 4 log (Figure 7B). This result is almost consistent with the results estimated with the disk diffusion test in Figure 6. The antibacterial ability of MRPs against *E. coli* was also reported by previous authors [3,14,17]. In the study of Rao et al. (2011) [17], the *E. coli* density after 24 h of treatment with a chitosan–glucose MRP was also decreased to 4 log CFU/mL. The obtained results revealed that the gamma ray-induced MR effectively improved the antibacterial activity of the CTS.

### 3.7. Evaluation of Cytotoxicity

**Figure 8:**
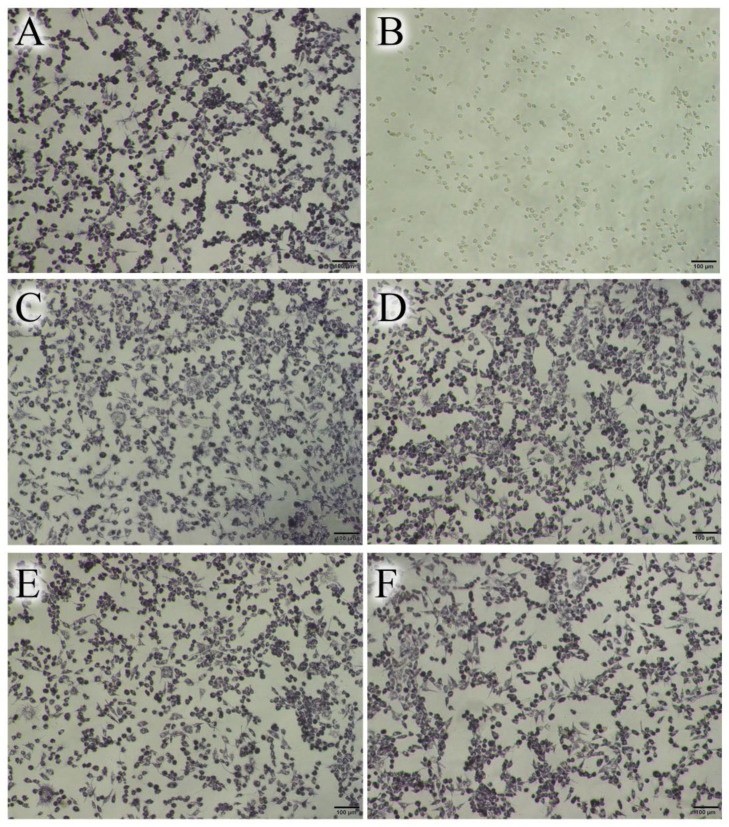
Microscopic images of L929 cells after 24 h of treatment with CTS-GA MRP at different concentrations. (**A**) Control (–); (**B**) positive control; and (**C**–**F**) CTS-GA MRP at 0.1, 0.25, 0.5, and 1.0 mg/mL, respectively.

The effect of the CTS-GA MRP concentration on the cell viability of the L929 cell line after incubation for 24 h is presented in Table 2. In the positive control, DMSO significantly reduced the cell viability compared to that of the negative control, in which the RGR reached 36.36%. On the other hand, there was no significant difference in the relative cell viability among different tested concentrations of the CTS-GA MRP. Moreover, the RGR of the CTS-GA MRP sample was also significantly high (>91%). Several authors have evaluated the toxicity of a compound as nontoxic, weakly toxic, or toxic when the relative cell viability is >70%, between 50% and 70%, or <50%, respectively [29]. Therefore, the result in Table 2 indicated that the CTS-GA MRP was non-cytotoxic in the range from 0.1 to 1.0 mg/mL. This result was also further confirmed by the microscope images of L929 cells treated with different concentrations of the CTS-GA MRP in Figure 8, where the number and morphology of treated cells were almost unchanged in comparison with the control. The low cytotoxicity of the MRPs of CTS was also recorded in other studies [22,26]. These results demonstrated that the MRP prepared with the gamma-ray irradiation method did not cause cytotoxicity.

## 4. Conclusions

In this study, a CTS-GA MRP solution was successfully prepared with the gamma ray-induced Maillard reaction at a dose of 25 kGy and freeze-dried to obtain a CTS-GA MRP powder. The as-prepared CTS-GA MRP powder exhibited good solubility in water and highly antibacterial activity against *E. coli* at a neutral pH. In addition, the obtained CTS-GA MRP was free from 5-HMF, an undesirable toxic byproduct. Furthermore, the CTS-GA MRP powder manifested non-cytotoxicity at a concentration of 0.1–1.0 mg/mL. Thus, the CTS-GA MRP can be used as a novel preservative for food and cosmetics. 

## Figures and Tables

**Table 1:** Absorbance at 284 nm and 420 nm of the CTS-GA solution before and after irradiation at 25 kGy and reaction efficiency.

	Absorbance at 284 nm	Absorbance at 420 nm	Reaction Efficiency (%)
Before irradiation	0.5540 ± 0.0353 ^a^	0.0868 ± 0.0088 ^a^	-
After irradiation	2.9612 ± 0.0278 ^b^	0.3695 ± 0.0314 ^b^	79.28 ± 1.79

The mean values in the same column with different letters are significantly different (*p* < 0.05) according to the Tukey test.

**Table 2:** Viability of L929 cells treated with CTS-GA MRP at different concentrations for 24 h.

Sample	Cell Viability (RGR %)
Negative control	100 ± 3.61 ^a^
Positive control	31.36 ± 4.55 ^b^
MRP CTS-GA 0.1 mg/mL	91.29 ± 4.58 ^c^
MRP CTS-GA 0.25 mg/mL	92.86 ± 3.02 ^c^
MRP CTS-GA 0.5 mg/mL	94.32 ± 2.69 ^c^
MRP CTS-GA 1.0 mg/mL	96.36 ± 2.76 ^c^

The mean values in the same column with different letters are significantly different (*p* < 0.05) according to the Tukey test.

## Data Availability

The data used to support the findings of this study are included in the article.
